# 终末期心力衰竭左心室辅助装置植入手术围手术期凝血管理4例并文献复习

**DOI:** 10.3760/cma.j.cn121090-20241128-00488

**Published:** 2024-12

**Authors:** 鹤昕 王, 洪武 王

**Affiliations:** 泰达国际心血管病医院麻醉科，天津 300457 Department of Anesthesiology, TICH, Tianjin 300457, China

**Keywords:** 心力衰竭, 终末期, 左心室辅助装置, 体外循环, 围手术期, 凝血功能, Heart failure, End-stage, Left ventricular assist device, Cardiopulmonary bypass, Perioperative period, Coagulation function

## Abstract

**目的:**

探讨终末期心力衰竭左心室辅助装置植入手术的围手术期凝血功能的管理。

**方法:**

收集2023年9月至11月在泰达国际心血管病医院进行左心室辅助装置植入手术的终末期心力衰竭的4例患者临床资料，包括手术情况、凝血指标、血制品与凝血药物的应用等，并作文献复习。

**结果:**

4例患者进行了左心室辅助装置植入手术，同期还进行了三尖瓣成形术和左心耳缝闭术，其中1例患者同期进行二尖瓣成形术、1例患者同期进行主动脉瓣置换术。所有患者在术中使用了血小板、冷沉淀等成分血制品和纤维蛋白原、重组人凝血因子Ⅶa等凝血药物。返回重症监护病房后所有患者接受了口服华法林和肝素桥接的抗凝治疗。患者未出现凝血相关并发症，均顺利出院。后续患者生存质量良好。

**结论:**

终末期心力衰竭左心室辅助装置植入手术的凝血管理受到病因、手术打击等多因素的影响，本文提出的管理策略可作为临床上减少凝血相关并发症的参考方法。

## Abstract (en)

**Objective:**

Exploring the management of perioperative coagulation function in patients with end-stage heart failure undergoing left ventricular assist device implantation surgery.

**Methods:**

Collect clinical data of four end-stage heart failure cases who underwent left ventricular assist device implantation surgery at TEDA International Cardiovascular Hospital from September to November 2023, including surgical conditions, coagulation indicators, application of blood products and coagulation drugs, and conduct literature review.

**Results:**

Four patients underwent left ventricular assist device implantation surgery, as well as tricuspid valve shaping and left atrial appendage closure surgery. One patient underwent mitral valve shaping surgery, while the other underwent aortic valve replacement surgery. All patients used blood products such as platelets and cryoprecipitate during the surgery. They also used clotting drugs such as fibrinogen and recombinant human coagulation factor VIIa during surgery. After returning to the intensive care unit, all patients received oral warfarin and heparin bridged anticoagulant therapy. All patients did not experience any coagulation related complications and were discharged smoothly. Currently, all patients have a good quality of life.

**Conclusion:**

The coagulation management of left ventricular assist device implantation surgery for end-stage heart failure is influenced by multiple factors such as etiology and surgical impact, and The management strategy presented in this paper can serve as a reference method for reducing coagulation-related complications in clinical practice.

终末期心力衰竭（end-stage heart failure，ESHF）严重影响患者生存，在心脏移植供体受限的情况下，其治疗越来越倚重左心室辅助装置（left ventricular assist device，LVAD）[1]。然而此类患者LVAD植入后敏感的容量限制和复杂的凝血管理形成了手术治疗的一大挑战[2]。为了探索ESHF患者LVAD植入手术围手术期合理凝血管理方案、减少手术后凝血相关并发症，从而加速患者术后康复，我们回顾了本中心4例ESHF患者LVAD植入手术的围手术期病历资料、手术情况、凝血指标和血制品与凝血药物的应用等，并作文献复习。

## 病例与方法

1. 病例：收集天津泰达国际心血管病医院2023年9月至11月收治的4例行LVAD植入手术的ESHF患者，回顾其围手术期凝血相关的病历资料，化验检查和影像学检查数据以及围手术期血制品使用的规律与临床结果。所有临床数据的采集与使用均在术前获得患者及家属的知情同意。

2. 抗凝管理：①术前抗凝：住院期间患者均每天1次皮下注射依诺肝素4 000 u，手术当天停用。②术中抗凝：肝素按400 u/kg静脉注射，体外循环期间维持激活全血凝固时间（ACT）在420～500 s，追加肝素量按1 u·kg^−1^·s^−1^计算。

3. 凝血管理：体外循环停机后静注鱼精蛋白中和肝素，按首次全量肝素1∶0.8给予鱼精蛋白，后续鱼精蛋白中和按1∶0.4的剂量在1 h内持续追加。首剂量鱼精蛋白中和后结合临床情况立即给予适量血制品与促凝血药物，随后进行凝血四项检查。根据凝血四项检查结果和临床情况再次针对性调整凝血功能，关胸后血栓弹力图检查凝血功能。

4. 输血标准：术中血制品使用标准根据《中华人民共和国卫生行业标准WS/T 796—202围手术期患者血液管理指南》执行。

## 结果

1. 临床资料与手术术式：4例ESHF患者，其中男3例、女1例，年龄42～69岁。4例患者出现心衰症状13～20年，均诊断为扩张型心肌病，术前机械循环支持机构间注册登记（Interagency Registry for Mechanically Assisted Circulatory Support，INTERMACS）分级均为2级，因卧床，术前均未行6分钟步行试验（6-minute walk test，6MWT）。其中例1有20年饮酒史，例3右下肢静脉曲张5年，例4 15年前行阑尾手术史。4例患者术前一般情况，心脏超声结果和凝血功能见表1。所有患者均行体外循环下LVAD植入术、三尖瓣成形术（TVP术）、左心耳缝闭术，其中例2同期行二尖瓣成形术（MVP术）、例3同期行主动脉瓣置换术（AVR术）。体外循环期间相关指标见表2。

**表1:** 4例终末期心力衰竭患者术前心脏超声与凝血指标

例号	性别	年龄（岁）	体重（kg）	血常规	凝血四项	NT-proBNP（ng/L）	LVEF（％）	FAC（％）	TAPSE（mm）
1	男	42	82	WBC：7.1×10^9^/L HGB：149 g/L PLT：181×10^9^/L	Fbg：4.47 g/L PT：12.6 s PT.INR：1.07 APTT：29.5 s TT：15.7 s	3 342	21	12	10
2	男	49	64	WBC：5.6×10^9^/L HGB：151 g/L PLT：276×10^9^/L	Fbg：5.88 g/L PT：12.6 s PT.INR：1.07 APTT：28.8 s TT：15.6 s	4 896	24	15	13
3	男	69	58	WBC：4.0×10^9^/L HGB：158 g/L PLT：179×10^9^/L	Fbg：1.54 g/L PT：12.2 s PT.INR：1.04 APTT：33.8 s TT：16.6 s	3 619	33	41	16
4	女	45	53	WBC：6.2×10^9^/L HGB：152 g/L PLT：158×10^9^/L	Fbg：2.18 g/L PT：12.9 s PT.INR：1.10 APTT：34.0 s TT：16.1 s	3 249	30	38	16

**注** NT-proBNP：N末端B型利钠肽前体；LVEF：左心室射血分数；FAC：右心室面积变化率；TAPSE：三尖瓣平面收缩期位移；Fbg：纤维蛋白原；PT：凝血酶原时间；PT.INR：凝血酶原时间国际标准化比值；APTT：活化部分凝血酶原时间；TT：凝血酶时间

**表2:** 4例终末期心力衰竭患者体外循环手术相关指标

例号	手术术式	CPB时间（min）	阻断时间（min）	CPB血浆使用量（ml）	CPB超滤总量（ml）	停机COP（mmHg）
1	LVAD植入术+TVP+左心耳缝闭术	135	99	480	1 800	26.5
2	LVAD植入术+MVP+TVP+左心耳缝闭术	127	100	240	1 000	25.5
3	LVAD植入术+AVR+TVP+左心耳缝闭术	180	129	480	2 500	24.5
4	LVAD植入术+TVP+左心耳缝闭术	99	75	360	1 700	28.1

**注** LVAD：左心室辅助装置；TVP：三尖瓣成形术；MVP：二尖瓣成形术；AVR：主动脉瓣置换术；CPB：体外循环；COP：血浆胶体渗透压

2. 凝血管理与血制品输注：4例患者手术全程输注氨甲环酸，按负荷剂量20 mg/kg，维持剂量10 mg·kg^−1^·h^−1^给予。中和肝素方案为按首次肝素化剂量的1∶0.8进行鱼精蛋白中和，后续手术中按1∶0.4持续输注鱼精蛋白，总中和剂量为1∶1.2。中和后根据患者术前病情和手术情况首轮给予部分血制品和凝血药物。根据凝血四项检验结果进行第2轮输注血制品和凝血药物，并在出室前再次检测凝血四项和血栓弹力图以验证凝血功能调整结果。4例患者体外循环后的血制品使用情况与凝血功能见表3。

**表3:** 4例终末期心力衰竭患者体外循环后血制品使用与凝血功能指标

例号	首轮血制品	首轮促凝药物	凝血四项	第2轮血制品	第2轮促凝药物	凝血四项	血栓弹力图
1	血小板：2 u； 冷沉淀：10 u； 新鲜冰冻血浆：520 ml； 悬浮红细胞：4 u	纤维蛋白原：2g； 重组人凝血因子 Ⅶa：2 mg	Fbg：2.69 g/L； PT：15.4 s； PT.INR：1.32； APTT：34.7 s； TT：16.4 s	血小板：1 u； 新鲜冰冻血浆：520 ml； 回收红细胞：860 ml	重组人凝血因子 Ⅶa：2 mg	Fbg：2.91g/L； PT：15.1 s； PT.INR：1.32； APTT：32.9 s； TT：16.0 s	R：11.2 min； K：3.1 min； Angle：51.0°； MA：62.6 mm； CI：−2.8
2	血小板：2 u； 冷沉淀：10 u； 新鲜冰冻血浆：360 ml； 悬浮红细胞：4 u	纤维蛋白原：2 g； 重组人凝血因子 Ⅶa：2 mg	Fbg：2.50 g/L； PT：11.2 s； PT.INR：1.04； APTT：32.4 s； TT：15.3 s	新鲜冰冻血浆：240 ml； 回收红细胞：740 ml	重组人凝血因子 Ⅶa：1 mg	Fbg：2.97 g/L； PT：12.2 s； PT.INR：1.09； APTT：32.1 s； TT：15.5 s	R：9.0 min； K：2.0 min； Angle：61.8°； MA：64.6 mm； CI：−1.8
3	血小板：2 u； 冷沉淀：10 u； 新鲜冰冻血浆：680 ml； 悬浮红细胞：4 u	纤维蛋白原：3g； 重组人凝血因子 Ⅶa：4 mg； 凝血酶原复合物： 600 iu	Fbg：1.82 g/L； PT：11.0 s； PT.INR：0.93； APTT：39.8 s； TT：15.7 s	血小板：2 u； 冷沉淀：10 u； 新鲜冰冻血浆：600 ml； 悬浮红细胞：2 u； 回收红细胞：920 ml	纤维蛋白原：2 g； 重组人凝血因子 Ⅶa：2 mg	Fbg：2.55 g/L； PT：11.3 s； PT.INR：1.08； APTT：38.6 s； TT：15.1 s	R：7.1 min； K：2.4 min； Angle：59.2°； MA：52.2 mm； CI：−2.4
4	血小板：2 u； 冷沉淀：10 u； 新鲜冰冻血浆：480 ml； 悬浮红细胞：4 u	纤维蛋白原：3 g； 重组人凝血因子 Ⅶa：3 mg； 凝血酶原复合物： 300 iu	Fbg：1.61 g/L； PT：18.4 s； PT.INR：1.62； APTT：48.8 s； TT：16.7 s	新鲜冰冻血浆：600 ml； 回收红细胞：520 ml	纤维蛋白原：2 g	Fbg：2.25 g/L； PT：17.2s； PT.INR：1.44； APTT：39.3s； TT：16.1s	R：5.0min； K：1.5min； Angle：55.8°； MA：61.2mm； CI：0.2

**注** Fbg：纤维蛋白原；PT：凝血酶原时间；PT.INR：凝血酶原时间国际标准化比值；APTT：活化部分凝血酶原时间；TT：凝血酶时间；R：反应时间；K：凝固时间；Angle：角度：MA：最大振幅；CI：凝血指数

3. 术后凝血管理与患者转归：术后4例患者均送返重症监护病房（ICU），抗凝方案为每日1次通过鼻饲管口服华法林，根据术前患者CYP2C9和VKORC1基因多态性检测结果，确定华法林口服剂量3～5 mg/d，其中手术日口服量为1.5倍的负荷剂量。同时在返回ICU 6 h后桥接肝素抗凝，静脉泵注10 u·kg^−1^·h^−1^，目标ACT在180～190 s。肝素使用至术后第3天口服华法林抗凝达标后停用。患者ICU住院期间相关指标见表4。4例患者无术后二次开胸，2～4 d后拔出胸腔引流管，ICU住院6～15 d，均无凝血相关并发症，顺利康复出院。出院后患者每周均接受电话随访，后续随访4例患者出院状态良好，心衰症状完全改善，运动能力提高。外科切口与经皮线缆伤口皮肤无红肿渗血。

**表4:** 4例终末期心力衰竭患者在重症监护病房（ICU）的相关检测指标

例号	入ICU血常规	肝素化后ACT值（s）	术后24 h胸引液总量（ml）	术后24 h凝血四项
1	WBC：7.2×10^9^/L HGB：105 g/L PLT：126×10^9^/L	186～199	800	Fbg：3.35 g/L PT：15.3 s PT.INR：1.31 APTT：31.5 s TT：15.5 s
2	WBC：8.4×10^9^/L HGB：94 g/L PLT：105×10^9^/L	172～189	870	Fbg：3.69 g/L PT：17.0 s PT.INR：1.50 APTT：31.7 s TT：14.5 s
3	WBC：8.9×10^9^/L HGB：92 g/L PLT：131×10^9^/L	182～204	1 210	Fbg：3.79 g/L PT：12.8 s PT.INR：1.29 APTT：37.2 s TT：14.5 s
4	WBC：9.3×10^9^/L HGB：103 g/L PLT：98×10^9^/L	174～211	1 055	Fbg：3.34 g/L PT：15.6 s PT.INR：1.36 APTT：32.8 s TT：15.5 s

**注** ACT：激活全血凝固时间：PT：凝血酶原时间；PT.INR：凝血酶原时间国际标准化比值；APTT：活化部分凝血酶原时间；TT：凝血酶时间

## 讨论并文献复习

1. 终末期心力衰竭的外科治疗：LVAD提高了ESHF患者的生存率、生活能力以及生存质量，逐渐被用于心脏移植前的桥接治疗和终末治疗[3]。ESHF患者存在心脏扩大，左右心室扩张，同时经常存在心脏瓣膜的关闭不全，据文献报道[4]–[6]，合并三尖瓣的患病率为30％～50％、合并二尖瓣的患病率为40％～70％、合并主动脉瓣的患病率为20％。虽然在LVAD植入手术同期行心脏瓣膜修复手术的标准目前仍存在部分争议[7]，但同期行心脏瓣膜手术的LVAD植入手术，手术操作更为复杂，延长体外循环时间和手术时间，增加出血量。而体外循环会通过有创，非生理过程对凝血功能造成损伤，包括对血小板，凝血机制的损伤，引起炎性介质大量释放以及纤溶系统的激活[8]。同时，不合理的输血促凝则可能导致泵内血栓等并发症，文献[9]指出，出血和血栓是LVAD植入的常见并发症，在成人患者中的发生率高达30％。因此，此类合并瓣膜手术的LVAD植入手术的围手术期凝血功能管理更为复杂。

2. 复杂心脏外科手术用血的困难：由于心脏手术存在失血和体外循环后发生凝血障碍的双重影响等，心脏手术为外科手术中血液输注量和个体差异性最大的手术类型[10]。有国内研究[11]显示，体外循环心脏手术后患者出现凝血功能障碍的发生率高达86.6％。且随着输血量的增加，并发症的发生率和死亡率随之增加[12]。同时现有的成分输血策略和大量输血方案更多针对的是创伤、产科和骨科，而非体外循环下心脏手术[13]。这使得在避免并发症的前提下改善心脏手术体外循环后凝血功能障碍的难度进一步加大。有研究指出，与创伤输血时红细胞、血浆、血小板按照1∶1∶1比例输注相比，复杂心脏手术出血量较大的患者输注更高比例的血浆和血小板，可能减少终末器官功能障碍和降低死亡率[14]。但目前最佳的血制品输注比例仍不明确。另一方面，在手术中凝血功能的化验检查需要花费较长的时间，随着术中病情的变化，这一延迟的结果可能减弱其实际指导意义。因此在手术实践中，本中心结合临床情况，经验性地使用一些促凝药物和成分血制品，输注后进行凝血功能检查。并在之后根据检查结果，补充追加促凝药物和成分血制品，并在手术结束前再次检查凝血功能以检验患者返回ICU前的凝血情况。这样既能循证地使用成分血制品和促凝药物，也可加速手术进程，从而减少相关的并发症。

3. 心脏外科手术用血的现有经验：尽管ESHF患者术前进行强化综合治疗，其体内仍有严重的水钠潴留影响患者的凝血功能。因此，我们强调体外循环停机后，应用改良超滤技术（modified ultrafltration，MUF）增加超滤总量[15]。同时，为保证停机时血容量的充足，在体外循环中输注血浆以增加平衡超滤量也是一种有效的方法。而在体外循环结束后，应用鱼精蛋白中和肝素是改善凝血的关键一步。然而，过多的鱼精蛋白可通过损害凝血酶的产生从而起到抗凝作用，并增强纤维蛋白溶解[16]。因此，确定鱼精蛋白合适的中和比例十分重要。文献[17]指出，按照鱼精蛋白0.84∶1的比例中和肝素，可逆转肝素的抗凝作用。抗纤溶作用的药物在心脏手术中应用较为广泛，氨甲环酸以其较好的安全性得到普遍使用。有荟萃分析指出，氨甲环酸可以减少输血、围手术期失血和再次手术的风险，且不会增加死亡、主要终末器官并发症或血栓形成事件的发生风险[18]–[19]。关于心脏手术中氨甲环酸的使用剂量，一项包含了3 079例患者的国内研究[20]显示，负荷剂量30 mg/kg、维持剂量16 mg·kg^−1^·h^−1^和体外循环预充量2 mg/kg的较高剂量并未增加不良结局的发生率，同时能减少同种异体红细胞的输注量。富血小板血浆（platelet-rich plasma，PRP）采集是指将血浆中血小板浓缩到高于全血中血小板水平的方法。其血小板浓度可以达到患者自身血小板浓度的2到6倍。荟萃分析指出，体外循环前进行PRP采集，在肝素逆转后输注PRP可减少术后失血[21]。然而，对于ESHF患者而言，维持血流动力学稳定可能更为重要，全麻后应尽可能快而平稳地进入体外循环。如在短时间内采集PRP则会进一步加剧患者的血流动力学波动，因此我们并未选择在ESHF患者中应用PRP采集。近年来，纤维蛋白原或凝血酶原复合物等促凝药物的作用已得到较多认可[22]。然而关于纤维蛋白原应用时机和应用剂量尚未达成广泛共识。有研究[23]认为，无论体外循环的时间长短，在体外循环结束时补充纤维蛋白原的效果都不劣于冷沉淀。而在另一项离体实验[24]中发现，补充纤维蛋白原可以逆转体外循环心脏手术后的血小板功能障碍。重组活化凝血因子Ⅶ常用于复杂心脏手术后难以控制的出血，以期取得满意的止血效果。通常在常规凝血试验正常但仍然出血的情况下，可考虑经验性输注重组活化凝血因子Ⅶ[25]。

4. 左心室辅助装置的围手术期抗凝方法：平衡出血和血栓形成是LVAD植入后管理的挑战之一[26]。ESHF的患者左心室严重衰竭，在植入LVAD后主动脉瓣难以开放。这种近乎平流的新的循环生理会使毛细血管迂曲扩张，导致内脏出血[27]。另一方面，剪切力介导的血小板活化被认为与LVAD术后的血栓形成相关，必须给予抗栓治疗。本中心通过前期大量动物实验和临床实践发现在出血和泵内凝血这一对矛盾中，出血是主要矛盾[15]。因此本中心选择在术中体外循环后全力促凝，维持正常的凝血功能，减少胸引液量；术后经鼻饲管口服华法林，同时在返回ICU 6 h后桥接肝素抗凝至3 d后口服华法林抗凝达标这一围手术期抗凝方案。所有患者均在术前进行CYP2C9和VKORC1基因多态性检测，指导术后华法林剂量调整，此方法可以确保华法林抗凝效果准确有效[28]。这一方案减少了术后胸引液量，也未产生泵血栓，同时长期随访也证明了其良好的效果[15],[29]。
